# Frequency and Temperature-Dependent Space Charge Characteristics of a Solid Polymer under Unipolar Electrical Stresses of Different Waveforms

**DOI:** 10.3390/polym13193401

**Published:** 2021-10-03

**Authors:** Hanwen Ren, Qingmin Li, Yasuhiro Tanaka, Hiroaki Miyake, Haoyu Gao, Zhongdong Wang

**Affiliations:** 1 State Key Laboratory of Alternate Electrical Power System with Renewable Energy Sources, North China Electric Power University, Beijing 102206, China; rhwncepu@ncepu.edu.cn (H.R.); hygaoeee@ncepu.edu.cn (H.G.); 2 Tokyo City University, 1-28-1, Tamazutsumi, Setagaya-ku, Tokyo 158-8557, Japan; ytanaka@tcu.ac.jp (Y.T.); hmiyake@tcu.ac.jp (H.M.); 3 College of Engineering, Mathematics and Physical Sciences, University of Exeter, Exeter EX4 4QJ, UK; zhongdong.wang@exeter.ac.uk

**Keywords:** space charge, polyimide polymer, unipolar electrical stress, temperature, frequency

## Abstract

In this paper, we studied the space charge phenomena of a solid polymer under thermal and electrical stresses with different frequencies and waveforms. By analyzing the parameter selection method of a protection capacitor and resistor, the newly built pulsed electro-acoustic (PEA) system can be used for special electrical stresses under 500 Hz, based on which the charge phenomena are studied in detail under positive and negative DC and half-wave sine and rectangular wave voltages. Experimental results show that the charge accumulated in the polyimide polymer under DC conditions mainly comes from the grounded electrode side, and the amount of charge accumulated with electric field distortion becomes larger in a high-temperature environment. At room temperature, positive charges tend to accumulate in low-frequency conditions under positive rectangular wave voltages, while they easily appear under high-frequency situations of negative ones. In contrast, the maximum electric field distortion and charge accumulation under both half-wave sine voltages occur at 10 Hz. When the measurement temperature increases, the accumulated positive charge decreases, with a more negative charge appearing under rectangular wave voltages, while a more positive charge accumulates at different frequencies of half-wave sine voltages. Therefore, our study of the charge characteristics under different voltage and temperature conditions can provide a reference for applications in the corresponding environments.

## 1. Introduction

With the rapid development of the transmission and transformation grid, power electronic equipment has come to play a more important role in power operation. In contrast to traditional DC and AC power equipment, the insulation of electronic equipment usually has to deal with special electrical environments, including high-frequency rectangular wave and sine-like voltages [1,2]. Under a sine voltage at power frequency, traditional equipment can operate continuously for several years. In contrast, due to the coupling effect of high-frequency voltage and high temperature, the insulation of electronic equipment may deteriorate within 2 years [3,4,5]. Therefore, the effective evaluation of the insulation characteristics of electronic equipment has become a necessary to ensure its safe operation.

Under the coupling effect of electrical and thermal stresses, a great deal of space charge usually accumulates inside insulation, which can further lead to electrical tree development, electric field distortion, flashover, and breakdown phenomena [6]. The current research of the space charge inside insulation mainly relies on reliable measurement methods. Among the various measurement techniques used at present, the PEA method has been widely used in traditional DC and AC stresses at power frequency due to its simple structure and mature measurement technology. Many researchers have found that the voltage amplitude, waveform shape, and temperature conditions can directly affect the charge accumulation of insulating polymers [7,8,9]. Therefore, an evaluation of the charge characteristics under the voltage and temperature environments faced by the insulating polymer will be helpful for its future application and modification design for the corresponding environments. 

For the special electrical stresses withstood by the insulation of electronic equipment, researchers have carried out some comparative studies on the charge characteristics of different polymers. From the results shown in [10], it is evident that the charge accumulation and electric field distortion inside cross-linked polyethylene polymers under unipolar half-wave sine and rectangular wave conditions are more serious than those under DC conditions. Meanwhile, the frequency can directly affect the charge characteristics. For the research of the polyamide-imide (PAI) polymer, it has also been found that the problem of accumulated charge is more severe under some unipolar voltage situations than that under DC stresses, and voltage polarity can also influence the charge accumulation [11,12]. A further comparison of the charge characteristics of polyester-imide, PAI, and polyimide (PI) polymers under special voltages was carried out in [13], with PI samples showing less space charge accumulation under rectangular wave stresses at 50 Hz. However, at present an analysis of the charge characteristics of this polymer under the effect of voltage frequency, temperature, and voltage waveform conditions is still lacking. Meanwhile, the existing research mainly focuses on the common conditions with low voltage frequencies and temperatures. PI material has been widely applied as the supporting insulation for high-frequency power electronic transformers, motors, and other electronic equipment. A comprehensive measurement of its charge characteristics under special electrical and high-temperature environments will aid in our understanding of the actual operating state of equipment insulation.

Based on the background described above, we carried out an investigation of the space charge phenomenon inside PI polymers under different temperature and voltage conditions. In contrast to the traditional PEA design for DC and AC conditions at 50 Hz, the key electrical component in the PEA system for high-frequency voltage conditions was analyzed and designed. After analyzing the component selection in the PEA system for high-frequency voltage conditions, the charge and electric field distributions under negative and positive DC and rectangular wave and half-wave sine voltages were studied. The effect of three temperature conditions was also compared. The research results of this paper firstly show the charge evolution under special unipolar electrical stresses in the space charge research field, which could be used to guide the design and modification of the insulating materials used for electrical performance improvement. Thus, this should provide an important reference for insulation system design in the manufacturing of power electronic equipment.

## 2. The PEA System Applied for High-Frequency Voltage Conditions

**Figure 1:**
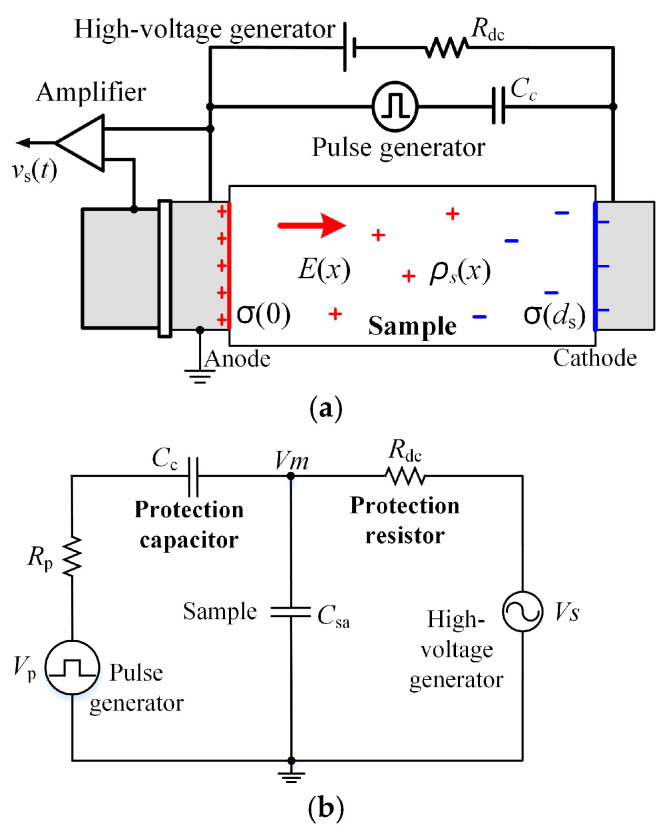
The PEA method for space charge measurement: (**a**) the measurement system, (**b**) the equivalent circuit.

The basic PEA system can be seen in Figure 1a. The space charge from the two electrodes was injected into the sample under the effect of electrical stress. The pulse voltage from the pulse generator was simultaneously imposed on the sample. Then, the pressure wave was generated from space charge due to its vibration and finally measured by the oscilloscope. The whole measurement processes was carried out via a computer [14].

Figure 1b shows the equivalent circuit of the PEA system based on the IEC Standard [15]. The values of protection resistor *R*_dc_ and capacitor *C*_c_ in Figure 1 can directly affect the voltage applied to the sample. According to the IEC standards, the protection capacitor should be smaller than 1 nF and the protection resistor should be larger than 10 kΩ. Based on this selection, the system has been widely used for DC or AC stresses at power frequency.

**Figure 2:**
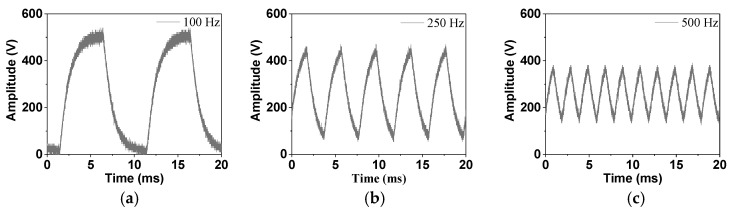
The voltages applied to the sample based on the traditional circuit design: (**a**) 100 Hz, (**b**) 250 Hz, (**c**) 500 Hz.

Since high-frequency voltages are studied in our research, the applicability of the traditional system was analyzed first. PI polymer was selected as the sample, and positive rectangular wave voltages with different frequencies were imposed on it. Figure 2 shows the voltage applied to the sample, as measured by a high-voltage probe. The amplitude of rectangular wave voltages was 500 V and the frequencies were 100, 250, and 500 Hz.

It can be seen from Figure 2 that the applied high-frequency voltage to the sample deforms obviously. The amplitude of the applied voltage decreases with frequency, which indicates that the system used for DC voltages cannot be applied under high-frequency voltages. Therefore, the system needs to be adjusted for use in this situation.

Based on the circuit of the PEA system shown in Figure 1b, the voltage *V*_m_ that was actually applied to the sample is represented by Equation (1).

(1) $$\left\{ \begin{array}{ll} V_{m} & =V_{s}-R_{\mathrm{dc}}\frac{V_{s}}{R_{\mathrm{all}}}\\ R_{\mathrm{all}} & =R_{\mathrm{dc}}+\frac{1}{j\omega C_{\mathrm{sa}}}⫽(\frac{1}{j\omega C_{c}}+R_{p})\\ & =R_{\mathrm{dc}}+\frac{j\omega R_{p}C_{c}+1}{j\omega C_{c}+(j\omega R_{p}C_{c}+1)j\omega C_{\mathrm{sa}}} \end{array}\right.$$

where *V*_s_ (V) represents the output voltage from a high-voltage generator. *R*_all_ (Ω) is the resistance of the whole circuit. *ω* (Hz) represents the angular frequency. *C*_sa_ (Hz) is the capacitance of the sample. *R*_p_ (Ω) is the resistance of the pulse generator.

**Figure 3:**
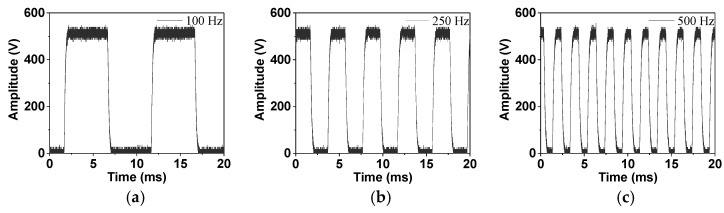
The voltages applied to the sample based on the improved circuit selection: (**a**) 100 Hz, (**b**) 250 Hz, (**c**) 500 Hz.

From this equation, the voltage applied to the sample can be calculated under the effects of the protection resistor and capacitor. Further, combined with the purchased components in the laboratory, we selected three protection capacitors of 560 pF in series and three protection resistors of 1 MΩ in parallel to establish a new system. The corresponding component values in the PEA system were 187 pF and 333 kΩ, respectively. This selection can decrease the probability of the flashover and breakdown of the components. Based on this design, the measurement of the voltage applied to the sample is shown in Figure 3.

Using this component selection, we can see that the voltages actually applied to the sample with different frequencies are consistent with the expected input—i.e., the voltages still maintain a rectangular wave shape and their amplitude does not deform. Therefore, we used this system for the charge measurements under unipolar voltage conditions. In addition, the selected components were only used for electrical stresses under 500 Hz in the later research. Therefore, the voltage applied to the sample is analyzed within 500 Hz to judge the applicability of the components. The above three frequencies, including 100, 250, and 500 Hz, clearly represent the waveform distortion of applied voltages. The results obtained from this new system design are also compared with those of the traditional system for DC voltages, which verifies that the new design also maintains a good accuracy.

**Figure 4:**
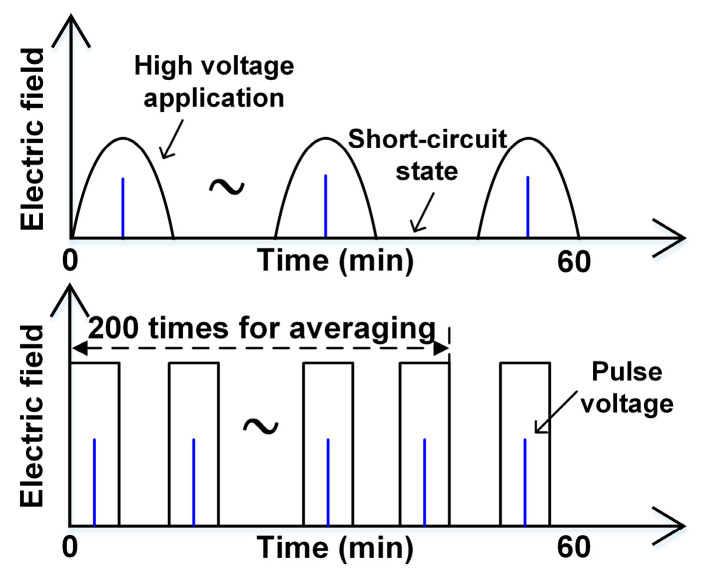
The measurement process for the two kinds of voltages.

For the studied DC voltages, a high voltage was applied to the sample for 30 min with a later 30-min short-circuit state, during which the charge measurement was carried out every 5 seconds. For the other voltage waveforms, Figure 4 shows the measurement method used for positive situations. The pulse excitation voltage was applied to the maximum amplitude of the half-wave sine voltages and the center point of rectangular wave voltages. The half-wave sine voltage contained only half a cycle of the sine waveform, while the voltage in the other half was zero. To ameliorate the signal-to-noise ratio of the measurement, 200 continuously measured signals were used for the averaging process. In the following measurements, the PI material usually used for electronic equipment was determined as the sample, whose thickness was about 125 μm. The amplitude of all the following applied electric fields was set as 60 kV/mm. In addition, for the inevitable distortion of the measured signal by the equipment, the corresponding calibration method was also designed, which also shows a high precision compared to past methods [14]. The measurement below was repeated many times to ensure that the results and conclusions were accurate and reliable.

## 3. Measurement Results under Different Voltage and Temperature Conditions

### 3.1. Results under Negative and Positive DC Stresses

**Figure 5:**
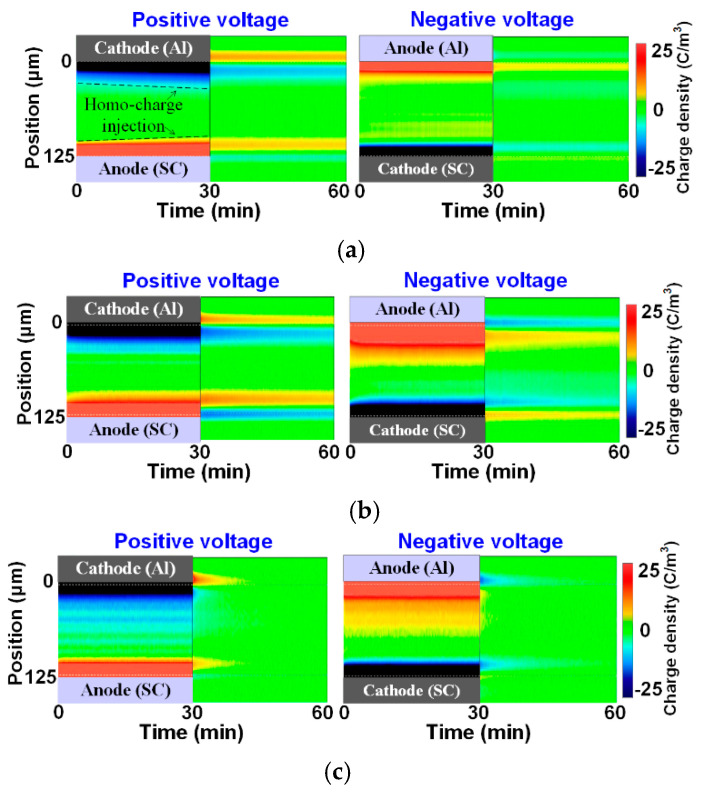
Space charge distributions under DC voltages: (**a**) room temperature, (**b**) 50 °C, (**c**) 80 °C.

The space charge characteristics under DC voltages with positive and negative polarities were firstly analyzed. Meanwhile, the effect of the three temperatures was also discussed, as shown in Figure 5. In the subfigures, the left part shows the voltage application state while the right part shows the short-circuit state when a high voltage was removed. The vertical axis represents the sample thickness direction from the grounded electrode to the upper electrode, while the horizontal one indicates the measurement time. The yellow and blue colors represent positive and negative charges, respectively.

From Figure 5, it can be seen that the polarity of the accumulated space charge inside the deep of the sample is the same as that of the grounded electrode. At room temperature, the charge injections from both of the electrodes could be found under the positive condition, while a small amount of positive charge accumulated in the polymer under the negative condition. When the temperature increases, more charge from the grounded electrode side migrates into the deep portion of the sample, indicating that the injection barrier at this side further decreases with temperature. From the results at the short-circuit stage, the extraction of space charge from the sample interfaces is slow at room temperature. However, the charge dissipates quickly from the electrodes after increasing the temperature, which means the extraction barriers of the electrode interfaces decrease and the migration of a large amount of space charge becomes very quick.

On the basis of the above charge distributions, the electric field results can be calculated using Poisson’s equation, as shown in Equation (2):

(2) $$E(z)\;=\frac{1}{\varepsilon_{0}\varepsilon_{r}}\int_{0}^{d_{s}}\rho (z)dz$$

where *E*(*z*) (kV/mm) is the electric field distribution inside the sample. *z* (mm) represents the position. *ε*_0_ (F/m) is the vacuum dielectric constant and *ε*_r_ is the relative permittivity of the sample. *ρ*(*z*) (C/m^3^) represents the space charge distribution.

**Figure 6:**
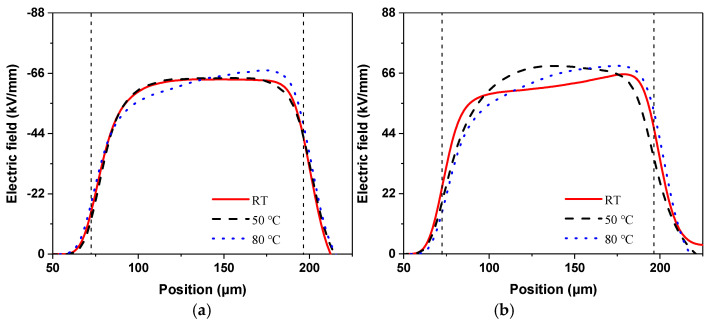
Electric field results under DC voltages: (**a**) positive voltages, (**b**) negative voltages.

Based on the above equation, the electric field distributions under DC voltages can be calculated, as shown in Figure 6. The results correspond to the last measurement of the voltage application state, which represents the field distribution along the direction of sample thickness. Under positive voltages, the electric field distributions found at room temperature and 50 °C are very close. The result obtained at 80 °C shows the maximum electric field distortion, whose value is about 67.09 kV/mm. Under negative voltages, the results at both 50 and 80 °C show high levels of electric field distortion. The maximum distortion also appears at 80 °C, the value of which is 68.64 kV/mm. Thus, the space charge accumulation and the electric field distortion are a little larger under the negative DC voltage application. This phenomenon also indicates that a higher temperature promotes space charge accumulation inside the PI sample. Meanwhile, combined with the results in Figure 5, charge accumulation and dissipation occur quicker under high-temperature conditions. This means that the electric field distortion could reach the states easily, as shown in Figure 6; thus, the material can be more easily damaged under high-temperature conditions.

### 3.2. Effect of Unipolar Rectangular Wave Voltages on Space Charge

**Figure 7:**
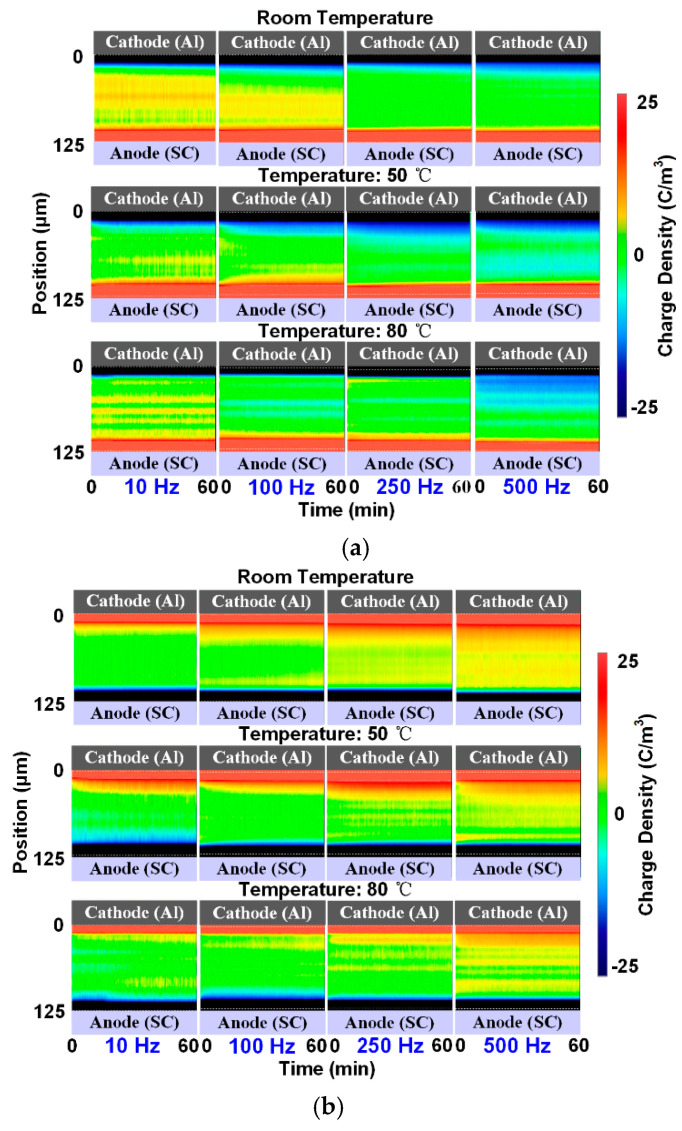
Charge results under rectangular wave voltages at different temperatures: (**a**) positive voltages, (**b**) negative voltages.

The charge phenomena occurring under positive and negative rectangular wave voltages within a frequency ranging from 10 to 500 Hz were then measured. Figure 7 shows the results obtained at room temperature, 50 °C, and 80 °C; the measurement time is illustrated in Figure 4. The three results shown in the columns correspond to one frequency, and the four results in the rows correspond to one temperature.

For the positive situations, positive charge accumulates inside the sample under the application of low-frequency voltage at room temperature from the upper electrode. At 250 and 500 Hz, barely any space charge appears in the deep of the material. At the higher temperature situation, it can be found that the amount of positive charge at the low-frequency voltage conditions decreases. In contrast, negative charge appears in the deep portion of the sample, especially at 500 Hz. This means that more negative charge accumulates under positive rectangular wave voltages when increasing the temperature, especially in high-frequency situations.

Similar to the results obtained under positive situations, positive charge appears in the sample under negative conditions at room temperature as well. However, the charge tends to accumulate under high-frequency conditions, especially at 500 Hz. When the temperature increases, the amount of positive charge decreases at each frequency condition. This phenomenon may be due to the same reason as that in the positive situation—i.e., more negative charge accumulates in the sample. The PEA measurement only presents the net charge distribution. Therefore, under the rectangular wave stresses with two polarities, the increase in temperature can cause a greater accumulation of negative charge when the amount of positive charge is decreased.

**Figure 8:**
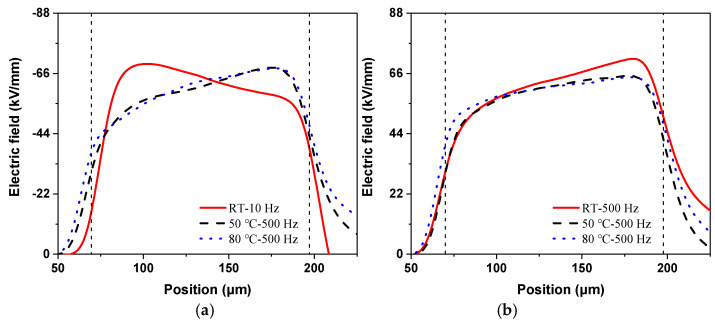
Electric field results under rectangular wave voltages at different temperatures: (**a**) positive voltages, (**b**) negative voltages.

From the above charge distributions, the electric field results can also be calculated at the last measurement of the voltage application, as shown in Figure 8. For simplicity, the most severely distorted electric field at each temperature condition is selected, as is also labeled in the figure.

Consistent with the charge results shown in Figure 7a, the maximum electric field distortion also appears at 10 Hz for the applied positive voltages, whose value is about 69.45 kV/mm. At 50 and 80 °C, the maximum field distortions are at the opposite site compared to the room temperature situation, which is due to the negative charge accumulation shown in Figure 7a. The two maximum distortions occur under the 500 Hz condition. Therefore, the increase in temperature reduces the electric field distortion at low-frequency situations and causes a more severe distortion in high-frequency conditions under positive rectangular wave voltages.

In contrast, the largest electric field distortions occur at the frequency of 500 Hz under negative voltages for each temperature condition. The maximum values of electric field at each temperature reach 71.32, 65.16, and 64.63 kV/mm, respectively. Therefore, the electric field distortion is alleviated a little with temperature due to the decrease in the positive charge, as shown in Figure 7b.

### 3.3. Results under Unipolar Half-Wave Sine Voltages

**Figure 9:**
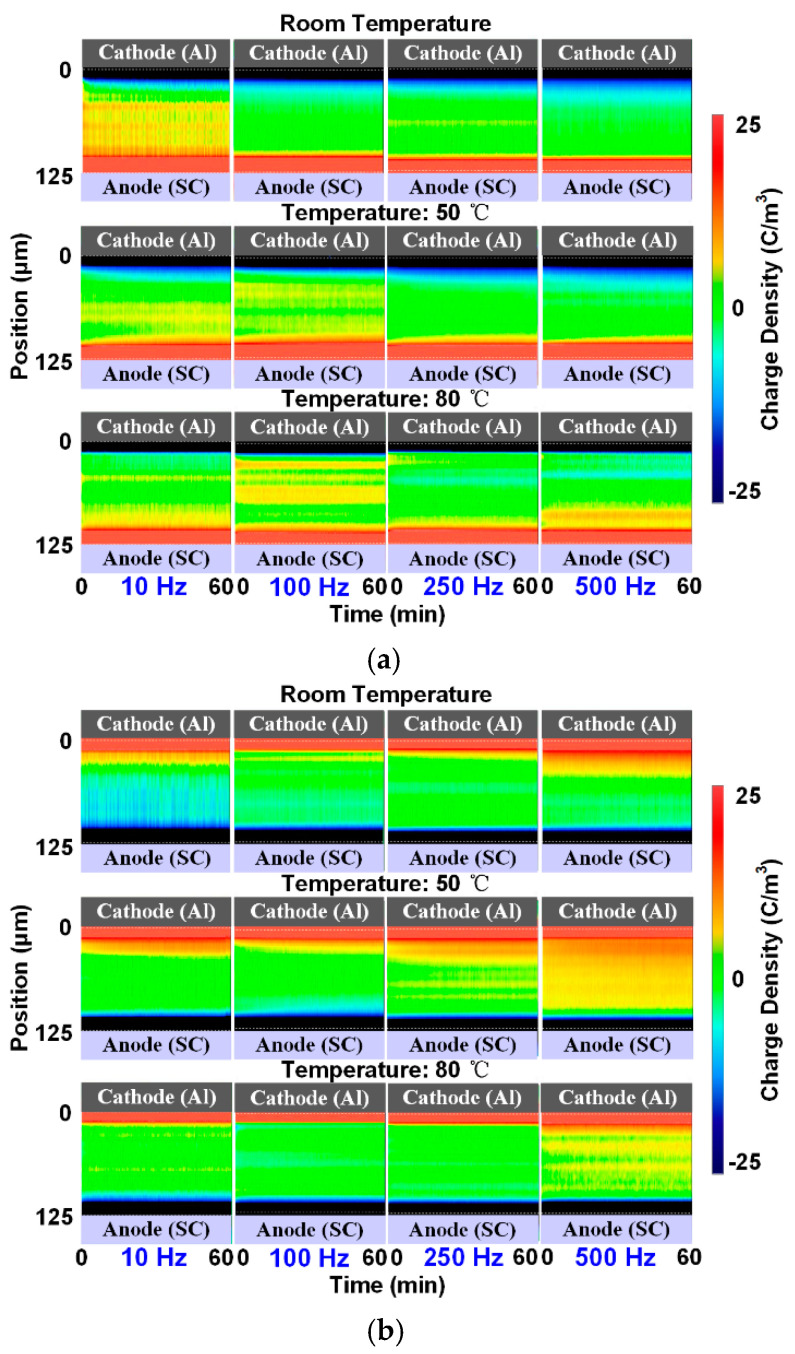
Charge results under half-wave sine voltages at different temperatures: (**a**) positive voltages, (**b**) negative voltages.

The results obtained under unipolar half-wave sine voltages were measured and discussed, as shown in Figure 9. The result at each condition corresponds to the maximum amplitude of the voltage waveform, as illustrated in Figure 4.

At room temperature, the space charge injected from the upper electrode layer accumulates in the deep portion of the sample under both the positive and negative voltages with a frequency of 10 Hz. When the voltage frequency increases, nearly no charge can be found in the deep portion, and only a small amount of charge from the two electrodes accumulates in their vicinity.

When the temperature increases, different charge phenomena are found. For positive situations, the amount of positive charge at the frequency of 10 Hz decreases, while the high-frequency situations including 100 and 500 Hz show some positive charge accumulation. In the negative voltage situations, barely any negative charge accumulates in the sample at 10 Hz compared to the room temperature condition. Meanwhile, a large accumulation of positive charge can be found at high-frequency conditions, especially at 500 Hz. Therefore, different from the results obtained under rectangular wave voltages, the increase in temperature causes a greater accumulation of positive charge under half-wave sine conditions with high frequencies.

**Figure 10:**
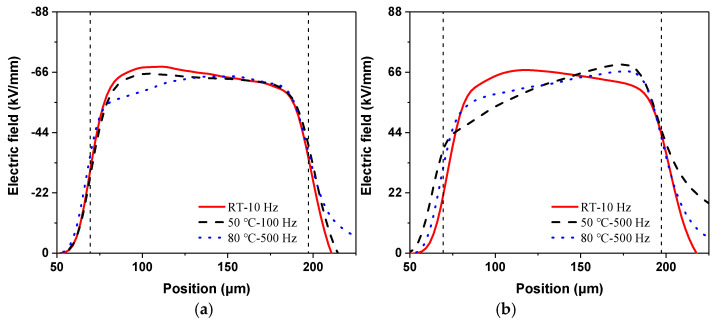
Electric field results under half-wave sine voltages at different temperatures: (**a**) positive voltages, (**b**) negative voltages.

Similarly, the electric field results calculated at the last measurement are shown in Figure 10.

For the positive conditions, the maximum electric field occurs under a condition of 10 Hz at room temperature, and the value is about 68.02 kV/mm. With temperature, the maximum electric field distortion appears at higher frequencies. In contrast, under negative voltage conditions, the maximum field distortion is found at the frequency of 500 Hz and the temperature of 80 °C, with a value of 68.82 kV/mm.

## 4. Discussion

From the above results, an interesting phenomenon can be seen; the charge accumulation is very quick under the conditions of special unipolar electrical stresses. The charge inside the deep portion of the polymer also dissipates quickly at the short-circuit stage of some DC conditions. This kind of rapid charge migration has also been found in previous charge measurements [16,17,18]. Under some DC and voltage transition conditions, space charge appears or dissipates immediately after the voltage state changes.

This phenomenon is caused by the rapid migration speed of space charge. In addition to the space charge with a mobility in the range from 10^−17^ to 10^−13^ m^2^/(Vs), which has been observed by most measurement results, the charge in shallow traps can migrate quickly inside the sample [19]. According to the space charge phenomena observed by some researchers, it has been calculated that the mobility of rapid charge is nearly 10^−9^ m^2^/(Vs) [20,21]. Meanwhile, some measured current results have also indicated that a part of space charge has a large migration speed [22]. From the analysis based on the results under the DC-superimposed pulsed voltage conditions [23], the charge inside the sample has been found to increase and decrease instantaneously after the pulsed voltage rises and falls. Therefore, this rapid charge migration exists under all kinds of voltage conditions. However, it is more obvious under special unipolar stresses due to the continuous and sharp changes in the applied voltage.

**Figure 11:**
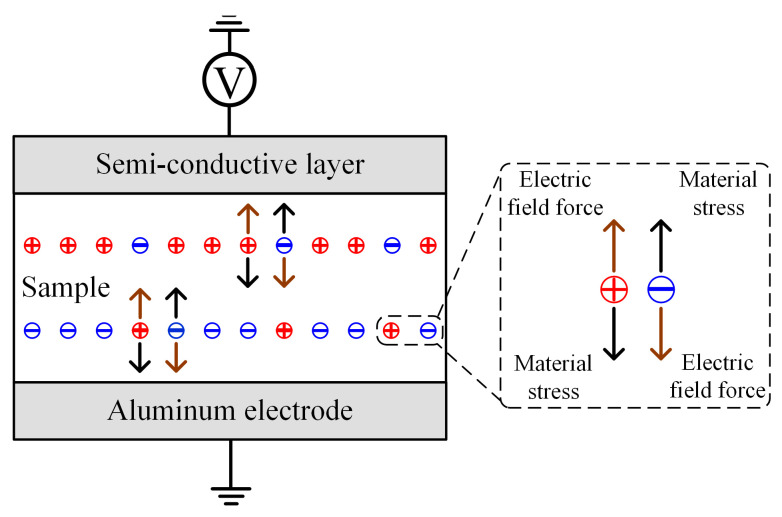
The forces withstood by the migrating space charge.

The charge accumulated inside the polymer withstands forces from many sources, which can generally be divided into two kinds—applied electric field force and the other force—as displayed in Figure 11 [23]. The former occurs due to the applied electrical stress. The latter mainly occurs due to the material itself, nearby charges, and other sources. Under stable voltage conditions, the forces withstood by the charge are also stable. Therefore, the charge can keep a relatively balanced state, can stay in traps for a long time, and have a low migration speed. In contrast, if the applied electric field continuously changes, the other force cannot always follow the applied electric field force due to the material deformation and relatively unchanged position between charges. Therefore, the charge obtains a faster migration speed more easily, due to the unbalanced force, and this phenomenon is more common under changing voltage conditions.

## 5. Conclusions

The space charge characteristics of PI polymer under different unipolar voltages and temperatures were studied. It was found that the accumulation process and evaluation law of charge are dependent on the voltage form, frequency, and temperature conditions. Some conclusions were obtained:

(1) The voltage that was actually applied to the sample was greatly affected by the protection capacitor and resistor in the PEA system. The circuit previously used for DC conditions is improper for high-frequency stresses, which can seriously deform the voltage applied to the sample when the frequency exceeds 100 Hz. After adjusting the protection capacitor and resistor to 187 pF and 333 kΩ, the PEA system could be used properly under 500 Hz.

(2) Experimental results achieved under DC voltages indicate that the space charge injected from the grounded electrode dominates in most cases. More charge accumulates inside the sample at a higher temperature, and the largest electric field distortions under the negative and positive stresses occur at 80 °C.

(3) In contrast to the charge phenomena under DC voltages, the results under special electrical stresses show frequency and temperature-dependent characteristics. For rectangular wave voltages at room temperature, positive charge accumulates easily under 100 Hz under positive conditions, while it accumulates more easily at higher frequencies under negative conditions. When the temperature increases, a negative net charge occurs under positive voltages, while the amount of positive charge is reduced under negative voltages, which indicates that an increase in temperature promotes the negative charge accumulation.

(4) For positive and negative half-wave sine voltages, the largest charge accumulations at room temperature occur at 10 Hz. When the temperature increases, a more positive charge is found inside the sample under different frequency conditions, which is completely opposite to the charge phenomenon that occurs under rectangular wave voltages. Meanwhile, the results under unipolar electrical stresses show the phenomenon of rapid charge accumulation. The special mechanisms of space charge accumulation and evolution under different voltages still need to be further researched.

## Data Availability

The data presented in this study are available on request from the corresponding author.
